# Assessment of Family Functioning and Eating Disorders – The Mediating Role of Self-Esteem

**DOI:** 10.3389/fpsyg.2019.00921

**Published:** 2019-04-24

**Authors:** Zdzisław Kroplewski, Małgorzata Szcześniak, Joanna Furmańska, Anita Gójska

**Affiliations:** Faculty of Humanities, Institute of Psychology, University of Szczecin, Szczecin, Poland

**Keywords:** eating disorders, self-esteem, family functioning, adulthood, mediation

## Abstract

The aim of the study was to measure whether people at increased risk for eating disorders (EDs) and people without an increased risk of EDs differ from each other in the assessment of family functioning (FF) and self-esteem (SE) dimensions. Moreover, the correlations between FF, EDs, and SE were verified, looking for the mediating role of SE in the context of the FF and EDs. The research was conducted on the group of 160 people aged from 18 to 47 years, including 74 people at increased risk for EDs. We used: The Family APGAR (Adaptability, Partnership, Growth, Affection, and Resolve); The SCOFF Questionnaire; The Multidimensional Self-Esteem Inventory, MSEI. Analyses indicate that the compared groups differ significantly in terms of EDs, assessment of FF, and all components of SE, in such a way that people without an increased risk of EDs are characterized by a more positive assessment of FF and higher SE in all its dimensions. All SE dimensions, except defensive high SE, are mediators in the relationship of FF with EDs. In therapeutic interactions, it is worth focusing on the SE dimensions, as they are one of the mediation elements in the relationship between the assessment of FF and EDs.

## Introduction

Eating disorders (EDs) are a group of disorders marked by abnormal eating attitudes which lead to significant disturbances in maintenance of healthy body weight (Le Grange et al., 2014; Rzońca et al., 2016), as well as serious and potentially long-lasting health problems, including death (O’Brien et al., 2017; Sadeh-Sharvit et al., 2018). Generally, EDs are first diagnosed before reaching adulthood (Striegel-Moore and Bulik, 2007), however, they may persist throughout a person’s life (Mangweth-Matzek et al., 2006; Hilbert et al., 2012; Tabler and Utz, 2015; Smink et al., 2018). In many cases, ED patients display instability of symptoms, switching from significant weight loss in adolescence to binging, and ceasing the purging behaviors in adulthood (Lavender et al., 2011). Moreover, non-diagnostic symptoms of EDs may occur in a broad population (Zarychta et al., 2017) and become more common among older persons (de Jong et al., 2018). The category of EDs includes both specific disorders such as anorexia (anorexia nervosa), bulimia (bulimia nervosa) and non-specific disorders (e.g., binge eating disorder or night eating syndrome) (Geliebter, 2002; Allison et al., 2005; Mitchison and Hay, 2014).

A review of the current literature on the subject indicates that both the etiology and the pathology of EDs are multifaceted (Latzer et al., 2009; McAdams and Smith, 2015) and still limited in understanding (McAdams et al., 2016). Researchers distinguish the biological (Striegel-Moore and Bulik, 2007; Weinberger-Litman et al., 2016; Haynos et al., 2018), individual (Loxton and Dawe, 2009; Farstad et al., 2016), cultural (Miller and Pumariega, 2001; Keel and Klump, 2003; Mayhew et al., 2017), and family factors as preventive or risk components in the development and maintenance of EDs (Denisoff and Endler, 2000; Leys et al., 2017). On the one hand, modern society is exposed to idealization of youth and the thinness presented in the mass-media (Thompson and Heinberg, 1999; Phelps et al., 2007; Striegel-Moore and Bulik, 2007). Several analyses (Murray et al., 1990; Eyal and Te’eni-Harari, 2013; Barcaccia et al., 2018; Ferguson, 2018; Griffiths et al., 2018) show that the body image represented on television, thinspiration/fitspiration websites, and magazine covers is commonly unrealistically thin for women or muscularity unideal for men, not reflecting the range of body shapes in the general population. On the other hand, biological predisposition and family related phenomena are also of significant importance as family provides grounds for comprehensive intellectual, emotional and social development, including personality and self-esteem (SE).

The multi-dimensional approach to EDs fits the diathesis-stress model of psychopathology. According to this paradigm, the development of EDs occurs as a result of the interaction of an individual’s biological or cognitive predispositions and severe environmental events (Hooley and Gotlib, 2000; Swearer and Hymel, 2015). Although the research literature on EDs is characterized by certain inconclusiveness (Balottin et al., 2017) as well as specific trends (von Ranson, 2008), various investigators report selected factors shared by both anorexia nervosa (AN) and bulimia nervosa (BN) patients. Individuals with AN and BN are often characterized by: perfectionism and scrupulousness (Joiner et al., 1997; von Ranson, 2008; Forsén Mantilla et al., 2017; Johnston et al., 2018), negative core beliefs about food and eating (Cooper and Hunt, 1998), body dissatisfaction (Troisi et al., 2006; Blodgett Salafia et al., 2015), self-criticism and low SE (Forsén Mantilla et al., 2017), alienation, shyness and social withdrawal (Leonidas and Dos Santos, 2017), shame and guilt (Burney and Irwin, 2000; Keith et al., 2009; Duffy and Henkel, 2016), anxiety (Egan et al., 2013; Stewart et al., 2015), alexithymia (Montebarocci et al., 2006; Pace et al., 2015), and perception of worse forms of family functioning (FF) (Keery et al., 2006).

Family functioning, often used as an umbrella term (McClellan and Cohen, 2007), encompasses a range of family constructs that reflect the nature of family relationships, cohesion and adaptability, sharing of decision making, mutual growth and self-fulfillment, caring and loving relationships, the commitment to share space, time and wealth, disclosure (Smilkstein, 1978; Kennedy et al., 2015). Although relationship between EDs and parental bonding is complex and not always well-defined (Holtom-Viesel and Allan, 2014), the results of several studies suggest the importance of familiar factors in specific and non-specific disorders (Allen et al., 2014; Tetzlaff and Hilbert, 2014; Balottin et al., 2017). For instance, some researchers (Le Grange et al., 2010; Holtom-Viesel and Allan, 2014) notice that EDs families describe their functioning as worse and more dysfunctional than control families, though there is little evidence for a typical pattern of such functioning. Moreover, the cross-sectional findings (Troisi et al., 2006) indicate that perception of FF in its different dimensions may be one of the important factors implicated in the negative body image assessment both in groups with AN and BN. Mayhew et al. (2017) observe that specific family dynamics, such as overprotection, intrusiveness, emotional unresponsiveness, and cross-generational enmeshment are related to EDs. Examples of factors which may be conducive to EDs and significantly affect their course as well as prognosis are: abnormal family relationships, both between the parents themselves (Blodgett Salafia et al., 2014), as well as between the parents and the children (Franko et al., 2008), an avoidance and inability to resolve conflicts (Erol et al., 2007; Gonçalves et al., 2016), overdependence, a lack of hierarchy and the loss of boundaries between family members (Barnhill and Taylor, 2001), a low level of attachment combined with a lack of affection and indifference as well as high expectations from the parents (Le Grange et al., 2010; Gonçalves et al., 2016; Cerniglia et al., 2017), and a negative parent-daughter relationship (Balottin et al., 2017; Korotana et al., 2018).

Nevertheless, the perception or experience of unsatisfying family relationship does not always result in EDs as the development of abnormal eating attitudes and behaviors may involve other intermediary mechanisms jointly responsible for such a relationship (Canals et al., 2009). Some researchers (Wei et al., 2005) indicate that FF can be associated with EDs beyond the direct links, and there is a need to explore more complex relations between both variables within a diathesis-stress approach. In their review, Zachrisson and Skårderud (2010) suggest theoretical hypotheses to explain the relationship between insecure attachment and eating pathology through the existence of some indirect factors.

Much of what we know about mediators and moderators of FF and EDs is based on the studies conducted to explore: personality characteristics, the intolerance of uncertainty, maladaptive emotional regulation, comparison with idealized others, perceived weight-related pressure, body dissatisfaction, and resilience. For example, some researchers (Eggert et al., 2007; Münch et al., 2016) show that higher neuroticism and lower extraversion mediate the relationship between insecure-resistant attachment and disordered eating. Others suggest (Sternheim et al., 2017) that a set of negative beliefs about uncertainty and a tendency to react negatively to uncertain situations and events may function as a moderating or mediating factor between attachment styles and ED-related symptoms. van Durme et al. (2015) indicate that deficits in the ability to effectively cope with challenging emotions partially mediates the relationships between attachment avoidance/anxiety and EDs. Still other authors (Tasca et al., 2009), testing a structural equation model of the association between attachment insecurity and ED symptoms, allude that body dissatisfaction and negative affect are mediators between the two factors. Finally, recent studies (Leys et al., 2017) reveal that resilience mediates the link between family dynamics and the occurrence of EDs.

Consistent with a diathesis-stress model, FF may promote the expression of EDs via self-esteem which is considered a vulnerability factor (Vohs et al., 1999). The focus on the concept of self-esteem in the present research arises from the sociometer theory (Leary, 2005) that explains self-esteem as an indicator of one’s relational evaluation. Thus, self-esteem reflects people’s beliefs concerning how they are perceived and assessed by others. The value attributed to the self is one of the most widely investigated aspects which are associated with EDs (Dada et al., 2017). The higher the degree to which an individual regards their relationship with another person as important, the lower their tendency to engage in dysfunctional behaviors. In the context of FF, it can be assumed that people’s feelings about themselves may be affected by parental evaluations remembered or lasting from childhood. Accordingly, self-esteem changes in response to a wide range of real and/or imagined rejections. While experience of positive ratings received from parents helps in building and maintaining child’s or adolescents’ healthy self-esteem, a feeling of negative assessment may lead to self-esteem reduction. Moreover, some studies (Vohs et al., 1999; Bardone et al., 2000) show that low self-esteem interacts with other factors to predict bulimic symptoms. Fairburn (2008) and colleagues (Fairburn et al., 2008) highlight self-esteem as a common characteristic among patients who binge, and a typical trait proceeding the eating problems. A longstanding and persistent negative view of the self, rooted in past events or experiences undergone in the present, may cause extremely self-critical esteem, generalized from “a specific failure” to “a general failure.” Therefore, low self-esteem appears to increase the risk of overvaluation of shape/weight, which in turn may lead to unhealthy weight-control practices, and consequently to eating-related psychopathology (Grilo, 2013; Pearl et al., 2014). A negative family history, reflected through a lack of warmth, unpredictability, criticism, overprotection and other adverse experiences may influence person’s dichotomous appraisals of self-worth and dysfunctional beliefs (Fairburn et al., 2008; Dalle Grave, 2013). Unstable identity is related to poor self-concept (fragile self-esteem and self-confidence), transferring to a major risk of EDs (Amianto et al., 2016). In turn, other studies provide preliminary and novel support that both mindful parenting skills and levels of self-compassion in adolescents help girls in the process of managing feelings of body shame and eating behaviors (Gouveia et al., 2018).

Additionally, transgenerational theories indicate that patterns transferred from one generation to another concerning, among others, intimacy models, autonomy, the system of values and coping skills have a direct influence on the functioning of the family – both as a unit as well as its individual members (Attili et al., 2004; Pilecki et al., 2014). Early experiences within a family context lead to the development of internal working models (IWM), theorized as beliefs in the self and others (Suzuki and Tomoda, 2015). A perception of parental dysfunctions toward oneself, such as inconsistency, intrusiveness or unresponsiveness, may result in an insecure IWM that activates some less functional strategies in dealing with anxiety (Attili et al., 2004). As parental attitudes have a long-term impact on a person’s functioning not only in childhood, but also in adulthood (Eddy et al., 2017), it is plausible to assume that internalization of an ambivalent or avoidant attachment style, may affect ones’ self-esteem, which can lead to depressive symptoms and EDs (Suzuki and Tomoda, 2015). In fact, current research (Chopik et al., 2013; Konrath et al., 2014) has confirmed that the impact of early childhood environments continues long after these bonds are formed, predicting the occurrence of the relationship dynamics after young adulthood and middle-age. Moreover, on the basis of a 7-year longitudinal study, Calam and Waller (1998) claim that the early teenage years seem to be crucial in the development of many women’s and men’s eating patterns in adulthood. Scabini et al. (2006) calls this process the transition to adulthood and affirms that the transition and its successful outcome strongly depend on the quality of family relationships.

Based on the former literature review, it could be argued that attention should be paid to individual characteristics and their mediating role with respect to the relationship between EDs and FF. Indeed, parent-child attachment seems to be associated with better treatment outcomes for EDs if patients receive therapy that promotes self-reflection (Tasca et al., 2009). The focus has been primarily on self-esteem as the key element of personality dimensions. A higher level of self-esteem seems to be connected with greater life satisfaction, activity, perseverance, optimism, and better coping with difficulties and loneliness. On the other hand, a lower level correlates with frequent experiencing of negative emotions (Geller et al., 2000), having difficulties with establishing social connections (Feixas et al., 2010), being more vulnerable to day-to-day incidents (Gila et al., 2005), seeing difficulties as insurmountable obstacles, an avoidant attitude toward problems and challenges (Martyn-Nemeth et al., 2009) as well as the repeated occurrence of psychosomatic symptoms (Mazzeo and Espelage, 2002; Łaguna et al., 2007).

In the context of the literature on the subject, the following assumptions were made: there is a difference regarding FF between persons with an increased risk to ED and those not suffering from such disorders (without ED) (hypothesis 1); the aforementioned groups differ in all dimensions of self-esteem (hypothesis 2); there is a positive correlation between FF and the dimensions of self-esteem accompanied by a negative correlation between ED and FF, as well as ED and self-esteem dimensions (hypothesis 3); there are dimensions of self-esteem which mediate the relationship between FF and ED (hypothesis 4) – thus providing a possible explanation why only some individuals with a negative assessment of the relationships in their family of origin suffer from an ED.

While hypotheses 3 and 4 are useful with a potential meaningful contribution to the literature, as only a few studies have analyzed how the perceived interpersonal relationships are related to EDs through the co-occurrence of other psychological variables (Pelletier Brochu et al., 2018), hypotheses 1 and 2 are very well-known already. However, the selection of self-esteem and FF as differentiating variables between the study and control groups (to verify 1st and 2nd hypotheses) was based on the transdiagnostic theory of EDs, in which it is assumed that a dysfunctional evaluation of self-worth and interpersonal problems are crucial in all types of EDs (de Jong et al., 2018). Moreover, the vast majority of studies concerns predominantly adolescent participants. Conversely, the aim of the present research was to reach older respondents representing early and middle adulthood.

## Materials and Methods

The study was conducted on 160 persons (80% women) in the age range of 18–47 (*M* = 23.18; *SD* = 5.06). The respondents were recruited from a social networking service dedicated to setting up ED support groups (Foundation “Światło dla Życia” – one of the first foundations in Poland that offer professional help to women and men with an ED, “Szklane Motylki” – a social network where individuals with ED problems associate themselves to exchange their ED experiences; the Standing Committee on Public Health – SCOPH – where EDs is one of the most important areas of concern). The low-risk control group (*N* = 86) was selected based on their score in the SCOFF Questionnaire (Sick, Control, One, Fat, and Food) (<2). The study group (*N* = 74) was chosen as being at risk of an ED based on the established SCOFF cut-off of ≥2 positive answers (Solmi et al., 2015). Those persons who were willing to participate in the study were contacted via a private message and provided with a battery of questionnaires, preceded with a specific instruction. The choice of the persons at increased risk for EDs through a social means was driven by the assumption that many individuals with anorexia, bulimia, and other non-specific disorders do not seek or receive treatment from specialist services (DeJong et al., 2013; Mitchison and Hay, 2014), and their disorder is not highly visible to others. The recruitment was done from a community sample, including women and not adolescents. The use of a non-clinical sample was chosen to add some new information; as previous research with the use of such groups have found some inconsistent outcomes (Mazzeo and Espelage, 2002).

The study group’s median age was 21 years with a range of 18–43 (69% women), and the control group’s median age was 23 years with a range of 18–47 (93% women). The control group was matched as closely as possible to the study group, according to appropriate continuous variables. While sex (control group: 68%; study group: 93%; *p* = 0.001), age (control group: *M* = 24.57; *SD* = 5.635; study group: *M* = 21.57; *SD* = 3.738; *p* = 0.001), and status of studying/working (*p* = 0.004) were different between both groups, the place of residence (*p* = 0.995) was comparable. Participants were not asked for detailed information about body mass index (BMI) as BMI is presently required for anorexia and not for other EDs (Gianini et al., 2017). Although the weight criterion used to diagnose EDs should be grounded in BMI (Hebebrand et al., 2004), more recent studies give no evidence (Machado et al., 2017) or modest support (Dakanalis et al., 2018) for the new DSM-5 severity ratings based on BMI level.

All of the respondents were assured of the confidentiality of their information and gave informed and written consent for their participation in the study. The research project was approved by the Bioethics Committee of the Institute of Psychology at the University of Szczecin (KB 03/2018).

### Method

For the purpose of verification of the hypotheses, the following were used: The Family APGAR (Adaptability, Partnership, Growth, Affection, and Resolve Scale) by Smilkstein et al. (1982), The SCOFF Questionnaire by Morgan et al. (2000), The Multidimensional Self-Esteem Inventory, MSEI by O’Brien and Epstein (1988).

Family APGAR is a brief screening tool used for a quick assessment of 5 dimensions of FF: adaptability, partnership, growth, emotions, and satisfaction with the time spent with the family (Namysłowska et al., 2000). Each of the 5 statements is assessed by the respondent in terms of the extent to which the person agrees with it – the scale ranges from 0 points (hardly ever) to 2 points (almost always). The lower the score, the greater the indication of a dysfunctional family system. Cronbach’s α reliability for the test proved to be satisfactory and amounted to 0.879 in the current sample. A single factor structure of the questionnaire (KMO measure of sampling adequacy 0.851; 67.522% of the explained variation) was also confirmed. Family APGAR correlates with other indices of FF, such as the Pless-Satterwhite FF Index (Ko et al., 2015), and psychological and physical health outcomes (Wu et al., 2010; Hai et al., 2017). A more negative evaluation of the family of origin is associated with higher loneliness (Wu et al., 2010) and fearful attachment (Diehl et al., 1998). Conversely, higher scores on the secure attachment style correlate positively with Family APGAR (Diehl et al., 1998).

The SCOFF Questionnaire is a popular and highly effective tool used in the United States and Western Europe, and increasingly more often in Poland, for the purpose of the identification of eating habits related to anorexia and bulimia (Morgan et al., 1999, 2000; Tsong and Smart, 2015) not only in clinical settings, but in the general population as well (Solmi et al., 2015). It is easy to apply and score (e.g., “Do you make yourself sick because you feel uncomfortably full?”; “Do you believe yourself to be fat when others say you are too thin?”), as consists of 5 questions to which the respondent gives either a positive “yes” or negative “no” answer. The number of “yes” responses is computed. The questionnaire revealed a single factor structure (KMO = 0.726; 43.431% of the explained variation). The respondents who scored 2 or more points were classified as belonging to the group of increased risk of EDs. In the present research, the average SCOFF score in the study group was *M* = 2.97 (*SD* = 0.09), while in the control group, it was *M* = 0.46 (*SD* = 0.50). Moreover, the diagnostic accuracy of the SCOFF was measured in order to assess its validity. The area of the receiver operating characteristic (ROC) curve, widely accepted as a method of selecting an optimal cut-off point for a test (Akobeng, 2007), showed moderate accuracy (0.730) with a 95% confidence interval of 0.653–0.807. The result is consistent with previous studies (Tsong and Smart, 2015). Therefore, it can be assumed that the SCOFF in the present study satisfactorily discriminates between individuals with a low-risk for an ED and those with a high-risk for an ED.

The MSEI is a 116-item self-report test. Respondents rate the items on a 5-point Likert scale: in the first part, the scoring is from 1 – strongly disagree to 5 – strongly agree, and in the second part from 1 – hardly ever to 5 – very often. The profile of the respondents concerns the following 11 scales: global self-esteem (satisfaction with the self and confidence), competence (ability to master new tasks), lovability (ability to express and receive affection), likeability (feeling accepted and liked by others), personal power (being assertive and able to influence others), self-control (being disciplined, persistent, able to set, and reach one’s goals), moral self-acceptance (satisfaction with one’s moral values and acting accordingly), physical attractiveness (being satisfied with one’s body image), vitality (motor coordination and the feeling of being fit), identity integration (the self’s internal integrity), and defensive high self-esteem (defensive reaction of increasing one’s self-esteem) (O’Brien and Epstein, 2009). Cronbach’s *alpha* for the entire scale showed high internal consistency and amounted to α = 0.966 in the present study. Eight of 10 subscales demonstrated Cronbach’s *alpha* equal or higher than 0.800: competence (α = 0.808), lovability (α = 0.825), likeability (α = 0.808), personal power (α = 0.800), self-control (α = 0.744), moral self-acceptance (α = 0.812), physical attractiveness (α = 0.863), vitality (α = 0.878), identity integration (α = 0.843), and defensive high self-esteem (α = 0.706).

The statistical analyses employed to verify the assumed hypotheses were conducted using IBM SPSS Statistics software, ver. 23. The statistics for Student *t* tests for independent samples were calculated, and the correlation analysis was done using Pearson’s *r* coefficient. A univariate covariance analysis was applied to assess whether the differences between the control group and the study group could also be accounted for by some sociodemographic data (sex, age, status of studying/working, and place of residence). The linear regression analysis was used to examine if and how much sex as a potential confounder in the model would distort the relationship between exposure and outcome variables.

**FIGURE 1:**
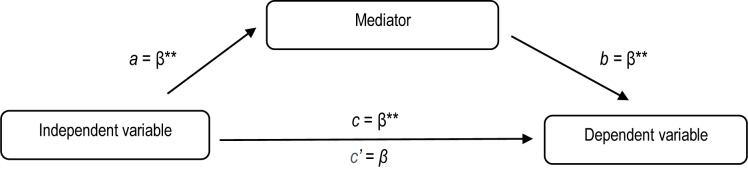
Theoretical model of the role of the mediator in the relationship between family functioning and eating disorders. ^∗^*p* < 0.05; ^∗∗^*p* < 0.01.

The PROCESS macro (version 3.2) was run to examine the indirect effects of each dimension of self-esteem separately on the relationship between FF and EDs. FF was the independent variable and the factor of EDs was the dependent variable. General self-esteem and all its ten components were mediating variables. Thus, there were eleven single-level mediation models no. 4 (Hayes, 2017), involving three-variable systems (Figure 1). For the current analysis, bootstrapping procedures were implemented. As recommended by Preacher and Hayes (2008), 5000 bootstrap samples and 95% confidence intervals were used to estimate the indirect effects that are considered significant if they exclude zero. This method appears to be superior to traditional mediation analyses because it does not require the data to adhere to assumptions of normality (Hayes, 2017).

## Results

According to the research aims, the first step was to check whether persons with an increased risk for ED (study group) and low-risk individuals (control group) show any differences in terms of the assessment of FF (hypothesis 1) and all dimensions of self-esteem (hypothesis 2). The results of the comparison – arithmetic mean (*M*), standard deviation (*SD*) for all of the analyzed variables, values of the Student *t*-test, significance level *p* and values of *d* Cohen are presented in Table 1.

**Table 1:** Family functioning and dimensions of self-esteem in the control group (without an increased risk for ED) and the study group (wih an increased risk for ED).

	Control group (*n* = 86)		Study group (*n* = 74)				
					
	*M*	*SD*	*M*	*SD*	*t*	*p*	*d Cohen*
Family functioning	6.058	2.754	4.513	3.093	3.341	0.001	0.527
Global self-esteem	27.988	7.579	21.445	7.376	5.512	<0.001	0.874
Competence	33.069	6.500	28.418	6.733	4.438	<0.001	0.702
Lovability	32.825	8.535	26.959	7.482	4.587	<0.001	0.730
Likeability	30.581	6.459	26.027	6.757	4.353	<0.001	0.638
Personal power	31.662	6.205	27.675	6.810	3.873	<0.001	0.612
Self-control	29.267	6.598	25.162	6.237	4.024	<0.001	0.639
Moral self-acceptance	37.872	6.093	32.918	6.505	4.969	<0.001	0.786
Physical attractiveness	27.872	7.992	22.527	7.467	4.347	<0.001	0.691
Vitality	29.825	7.654	24.121	8.272	4.469	<0.001	0.715
Identity integration	29.627	7.669	24.459	6.829	4.527	<0.001	0.711
Defensive high self-esteem	48.337	8.385	45.135	7.262	2.561	0.011	0.408


*^∗^*p* < 0.05*;*^∗∗^*p* < 0.01; Family functioning (APGAR); global self-esteem, competence, lovability, likeability, personal power, self-control, moral self-acceptance, physical attractiveness, vitality, identity integration, and defensive high self-esteem (MSEI).*

The results of the analysis conducted using Student *t*-test for independent samples show that there are statistically significant differences between the two groups in terms of increased risk for ED, FF and all of the components of self-esteem. In most cases, the strength of the effect measured with *d* Cohen is moderate in magnitude. Only in the case of defensive high self-esteem, the strength was found to be low. However, in the case of global self-esteem, the effect size was large. Individuals without an increased risk for ED are characterized by a more positive FF and higher self-esteem in all dimensions.

We also used an analysis of covariance to control for differences in EDs based on gender, age, and working/studying variables, as they resulted in differences between both groups (study vs. control). The results of the Levene’s test of equality of error variances indicated that the assumptions of homogeneity of variances were met (*p* = 0.472). The results show that age and working/studying were not significant covariates [*F*(1, 159) = 1.769; *p* = 0.186; partial eta squared = 0.012 for age; *F*(1, 159) = 2.344; *p* = 0.128; partial eta squared = 0.016 for working/studying]. Instead, gender was a significant covariate *F*(1, 159) = 8.948; *p* = 0.003; partial eta squared = 0.058.

The next step was to verify the relationship between an increased risk for ED, FF and dimensions of self-esteem (hypothesis 3). Pearson’s *r* correlation (Table 2) shows a negative correlation between all of the dimensions of self-esteem and an increased risk for ED and a positive correlation between the dimensions of self-esteem and FF.

**Table 2:** Correlation between an increased risk for ED, FF, and dimensions of self-esteem.

Dimension of self-esteem	Eating disorders	Family functioning
Global self-esteem	-0.402^∗∗^	0.413^∗∗^
Competence	-0.333^∗∗^	0.276^∗∗^
Lovability	-0.343^∗∗^	0.684^∗∗^
Likeability	-0.327^∗∗^	0.462^∗∗^
Personal power	-0.294^∗∗^	0.171^∗^
Self-control	-0.305^∗∗^	0.419^∗∗^
Moral self-acceptance	-0.368^∗∗^	0.381^∗∗^
Physical attractiveness	-0.327^∗∗^	0.400^∗∗^
Vitality	-0.339^∗∗^	0.430^∗∗^
Identity integration	-0.335^∗∗^	0.304^∗∗^
Defensive high self-esteem	-0.200^∗∗^	0.250^∗∗^


*^∗^*p* < 0.05*;*^∗∗^*p* < 0.01; family functioning (APGAR); eating disorders (SCOFF); global self-esteem, competence, lovability, likeability, personal power, self-control, moral self-acceptance, physical attractiveness, vitality, identity integration, and defensive high self-esteem (MSEI).*

In order to detect multicollinearity (Kim et al., 2007), the regression model was tested because of correlations among all of the dimensions of self-esteem. In both groups (a low-risk for ED, and an increased risk for ED), tolerance values ranged from 0.350 to 0.839 and the variance inflation factor (VIF) values ranged from 1.192 to 2.881, respectively, suggesting that multicollinearity was unlikely to be an issue in our study.

In the subsequent part of the analyses, dimensions of self-esteem were introduced as potential mediators which could weaken, strengthen or have no influence on the existing correlation between the independent variable (FF) and the dependent variable (an increased risk for EDs) (Figure 1).

The PROCESS macro shows (Table 3) that the c path (the direct effect) decreased after the introduction of 10 out of 11 mediators and became insignificant (c’ path). On the basis of the obtained results, it can be stated that global self-esteem, competence, lovability, likeability, personal power, self-control, moral self-acceptance, physical attractiveness, vitality, and identity integration mediate the relationship between FF and an increased risk for EDs. Defensive high self-esteem did not mediate the relationship between FF and increased risk for EDs, as evidenced by confidence interval for the indirect effect that contained zero (mediation model 11): 95% CI [-0.0387, 0.0025].

**Table 3:** The role of global self-esteem and its dimensions in the relationship between FF and an increased risk for EDs.

	a Path	b Path	c Path	c’ Path	Indirect effect	B(SE)	lower CI	Upper CI
1. FF – GS – ED	1.11^∗∗∗^	**-**0.07^∗∗∗^	**-**0.10^∗∗^	**-**0.02 (ni)	**-**0.0817	0.0211	**-**0.1263	**-**0.0434
2. FF – CO – ED	0.64^∗∗∗^	**-**0.04^∗∗^	**-**0.10^∗∗^	**-**0.07 (ni)	**-**0.0319	0.0144	**-**0.0628	**-**0.0073
3. FF – LO – ED	1.94^∗∗∗^	**-**0.07^∗∗∗^	**-**0.10^∗∗^	0.04 (ni)	**-**0.1479	0.0354	**-**0.2173	**-**0.0766
4. FF – LI – ED	1.07^∗∗∗^	**-**0.06^∗∗∗^	**-**0.10^∗∗^	**-**0.03 (ni)	**-**0.0719	0.0214	**-**0.1167	**-**0.0333
5. FF – PP – ED	0.38^∗^	**-**0.05^∗∗^	**-**0.10^∗∗^	**-**0.08 (ni)	**-**0.0213	0.0127	**-**0.0499	**-**0.0010
6. FF – SC – ED	0.93^∗∗∗^	**-**0.05^∗∗^	**-**0.10^∗∗^	**-**0.05 (ni)	**-**0.0530	0.0184	**-**0.0893	**-**0.0171
7. FF – MS – ED	0.85^∗∗∗^	**-**0.07^∗∗∗^	**-**0.10^∗∗^	**-**0.04 (ni)	**-**0.0626	0.0194	**-**0.1026	**-**0.0276
8. FF – PA – ED	1.08^∗∗∗^	**-**0.05^∗∗∗^	**-**0.10^∗∗^	**-**0.04 (ni)	**-**0.0599	0.0205	**-**0.1020	**-**0.0229
9. FF – VI – ED	1.20^∗∗∗^	**-**0.04^∗∗^	**-**0.10^∗∗^	**-**0.05 (ni)	**-**0.0516	0.0199	**-**0.0947	**-**0.0158
10. FF – II – ED	0.78^∗∗∗^	**-**0.05^∗∗∗^	**-**0.10^∗∗^	**-**0.06 (ni)	**-**0.0400	0.0150	**-**0.0727	**-**0.0142
11. FF – DH – ED	0.66^∗∗^	**-**0.01	**-**0.10^∗∗^	**-**0.09 (sig)	**-**0.0151	0.0106	**-**0.0387	0.0025


*^∗^*p* < 0.05; ^∗∗^*p* < 0.01; ^∗∗∗^*p* < 0.001; ni – non-significant; sig – significant; FF, Family functioning; ED, Eating disorder; GS, General self-esteem; CO, Competence; LO, Lovability; LI, Likeability; PP, Personal power; SC, Self-control; MS, Moral self-acceptance; PA, Physical attractiveness; VI, Vitality; II, Identity integration; DH, Defensive high self-esteem.*

All of the cases presented above (Table 3) reveal that FF is indirectly associated with an increased risk for EDs through self-esteem dimensions: general feeling of self-confidence, belief in one’s ability to perform new tasks competently, certainty of being loved and taken care of, feeling accepted by peers, feeling powerful and assertive, being self-disciplined and persevering, satisfaction with moral values and acting in accordance with them, feeling attractive to others, enjoyment of physical activities, and having a clear sense of identity and cohesion.

Respect to sex as a potentially confounding variable, it did not make a significant contribution to the model as a change in the regression coefficient was very small between measurement with (*R^2^* = 0.459) and without sex (*R^2^* = 0.429). However, in different equations and with different sets of independent or confounding variables this outcome could potentially change.

## Discussion

As has been assumed in hypothesis No 1 and 2, it was found that persons with no risk of EDs are characterized by a more positive assessment of FF and a higher level of self-esteem in all its dimensions as compared with individuals at risk of EDs. Such results are in line with the majority of the outcomes given in the literature on the subject which show that patients with EDs seem to hold more maladaptive core beliefs than their low-risk counterparts (Dingemans et al., 2006; Cerniglia et al., 2017). For example, Blodgett Salafia et al. (2015) present results supporting differences in the perception of various life domains between individuals with and without EDs. Those participants who were at risk of an ED tended, to a greater extent than those without an ED, to associate their low self-esteem with a bad relationship or social problems, listed traumatic life events as related to the beginning of their disorder, and mentioned poor body image as a foundation for the development of the ED. Other studies (Dingemans et al., 2006) demonstrate that ED patients have less functional thinking patterns related not only to diet, eating, weight or shape, but associated with their environment, including close family and friend relationships. Such maladaptive schemas often develop as a result of interpretation of present situations, memories from the past experience, or convictions about the future (Jones et al., 2007), resulting in less constructive behaviors, perceptions of the world and themselves, feelings, and interactions (Young et al., 2003). Among the main areas of dysfunctional cognitions of persons with diagnosed EDs, researchers (Legenbauer et al., 2018) mention negative thoughts about the persons’ own body and self-esteem.

The third hypothesis was also confirmed, showing a negative correlation between all dimensions of self-esteem and an increased risk for ED, and a positive correlation between dimensions of self-esteem and FF. While on the one hand parenting is associated with self-esteem and satisfaction, on the other hand, parental styles may also be related to abnormal attitudes (Liu et al., 2018). Researchers point to the negative traits of a family system (overprotectiveness, difficulties with resolving disagreements and problematic situations, distance between family members or an atmosphere of tension or misunderstanding) as associated with EDs (Namysłowska et al., 2000). The family environment may be reflected in the self-esteem of an individual suffering from an ED – most often it is unrealistic, low and strongly influenced by a feeling of guilt for experienced misfortune (Ziółkowska, 2005).

Verification of the proposed hypothesis No 4 seems to indicate that global self-esteem, competence, lovability, likeability, personal power, self-control, moral self-acceptance, physical attractiveness, vitality, and identity integration mediate the relationship between FF and EDs. The presence of self-esteem mediators explains why the negative assessment of the functioning of the family of origin does not necessarily translate into the development of EDs.

The obtained outcomes are in line with the results of some recent clinical analyses. Dissatisfaction or self-doubt can be a manifestation of a negative perception of oneself, thus constituting a high-risk factor of an unhealthy weight loss or the development and persistence of an ED (Noordenbos et al., 2014). Pelletier Brochu et al. (2018) report that low self-esteem and negative mood act as mediators of the level of perceived alienation in the relationship with an individual’s mother and ED symptom severity. The contrary situation is observed among individuals with a higher level of self-worth who tend to adopt a more positive attitude to events from the past and perceive the future as having a possibility for success, including overcoming an ED (Garcia et al., 2017).

In regard to competence as a mediator between FF and EDs, the previous research shows some similar outcomes. For example, self-competence seems to play a role in bulimic behavior. The findings (Gordon et al., 2005) suggest that middle-aged and older women who display low levels of self-competence and feelings of ineffectiveness tend to be more vulnerable to bulimic symptom development rather than assuming adaptive behaviors of weight loss. The reason for such choices may stem from their disbelief in their ability to change body weight in healthy way. Other researchers (Bardone et al., 2003) provide empirical support that self-competence is more predictive of the change in EDs over time.

Another significant mediating factor is the belief of being loved, even if such a belief stems not from the relationship with family members but from other people. According to Stice and Whitenton (2002), individuals at increased risk for EDs tend to feel a lack of unconditional support and emotional closeness from family members and friends, which predispose them to form a negative body image. Persons with an ED struggle to please others and, at the same time, to win their affection (Brown and Geller, 2006). In fact, Williams and Reid (2012), using an interpretative phenomenological analysis, report some evidence obtained from participant with EDs who wanted to recover from their disorder. One of the girls believed that she needed to be perfect, polite, and put others first to be liked and loved. Another young woman defined anorexia as an inner, constant, and controlling voice which criticizes who you are and what you do, reminding you that you are worthless and unloved. Still, another participant recognized that the conviction of being loved by other people motivated her to start defeating her disease. Such an admission shows that being aware of receiving affection can greatly increase the chances for successfully overcoming the disorder, as a person who feels loved manifests greater emotional strength and is less likely to suffer from depression.

With respect to personal power and self-control as mediators between FF and EDs, preliminary findings (Bandini et al., 2013) illustrate that low assertiveness can maintain symptoms and aggravate the outcome in patients with EDs. Other studies (Williams et al., 1990, 1993) show that ED individuals, contrary to normal controls and individuals who display dietary features, report significantly less self-assertion and more external control, thus supporting the anecdotal contentions that both low self-esteem and low assertiveness are traits of AN and BN patients. As both personal power and self-control denote a person’s ability to override and constrain socially intolerable and undesirable impulses and to modify and regulate one’s behavior (Finkenauer et al., 2005), such capacities may help in altering EDs. Lu et al. (2015) confirm that self-control serves as a mediator between parental overprotection and EDs.

Moral self-approval, which refers to satisfaction with the individual’s values and moral standards, is the next important mediating factor between FF and EDs. Similar results are presented by Collin et al. (2016) who found that moral self-approval is predictive of magnitude of change in the dimensions of an eating psychopathology, precisely of AN. Moreover, other outcomes show (Mulkerrin et al., 2016; Masuda et al., 2018) that individuals at increased risk for EDs, contrary to their low-risk counterparts, often experience difficulty in recognizing and refining their personal values. They tent to live with their values and principles in an extreme or incongruent way, assuming rigid, inflexible attitudes about some aspects of their lives (e.g.: own weight, appearance, dietery restrictions) or compromising interpersonal values of close family relationships and friendships by choices arising from EDs. Such unhelpful beliefs and inappropriate practices may be challenged and modified through psychological treatments, giving them a chance to defeat the desease.

The importance of feeling physically attractive in the development of EDs is reflected not only in the mediating role gained in the present study, but also by the results obtained previously (Davis et al., 2000; Patzer, 2006; Vartanian et al., 2018). Studies worldwide (Davis et al., 2000; Józefik and Pilecki, 2010) have found that exaggerating the value of appearance can trigger excessive concern for one’s own image, thus leading to unhealthy weight loss and, consequently, to the development of an ED. In fact, Goñi and Rodríguez (2007) highlight that the feeling of dissatisfaction with one’s body image may be one of the most significant predictors of EDs as a distorted body image often relates to a low physical self-concept. In analyses that have utilized multi-dimensional self-esteem instruments (O’Dea, 2006), a number of explicit domains of self-worth have been found to be negatively associated with body weight (e.g.: physical appearance and attractiveness, athletic competence).

Vitality known as the sense of being in good physical shape and being physically active, can have a positive impact on the negative correlation between the assessment of FF and EDs. When a person’s vitality is strong, it can serve as a major motivating factor for coping with difficult family situation and possible problems resulting from the disorder. The studies (Johnson and Wardle, 2005) show that people who are dissatisfied with their body image and are in poor physical shape are at an increased risk for EDs, both anorexia and bulimia. The negative emotions experienced by a person with low self-esteem can lead to an increased risk of a distorted body image (Reboussin et al., 2000), which in turn results in taking deliberate action aimed at ongoing weight loss and all its physical consequences.

Finally, identity integration appears to be another essential factor related to EDs. This result is in line with other previous studies. For example, Fairburn and Harrison (2003) hold that individuals with EDs are inclined to overvalue their body shape and/or weight. Moreover, Stanghellini et al. (2012) consider that the different types of irregularities characterizing persons with an increased risk of EDs are consequences of their personal identity.

The present research extends our knowledge about the mediatory role of self-esteem and its dimensions between FF and EDs, explains why only some individuals with a negative assessment of the relationships in their family of origin may display EDs, and offers some conclusions that have potential clinical implications. However, the present investigation also has some limitations. As the current analyses were based on a subset of the small group of participants and had a cross-sectional character, it is impossible to establish causal relationships between variables, and the analyses should be considered cautiously without generalization. In fact, mediation with cross-sectional data does not tell us to what extent the association between self-esteem and EDs reflected an actual effect of the self-esteem on ED symptoms, and to what extent the associations resulted from those with lower-risk EDs being more confident and satisfied with the self. Furthermore, the constructs of FF, self-esteem, and EDs were measured through self-reports and, in the case of FF, were formed on retrospective data referring to the individual’s childhood. Although some studies demonstrate encouraging levels of consistency between retroactive and contemporaneous narratives (Brown, 2014), the accuracy of retrospective reports is sometimes considered problematic because of potential memory bias and, consequently, is believed to not always be sufficiently valid (Naicker et al., 2017; Bell and Bell, 2018). Moreover, it could be argued that the family system across the sample varied widely given that some individuals in the upper age bracket were in middle adulthood and might be parents themselves, whereas individuals in the younger bracket were entering adulthood and did not have experience of parenthood. As such, the results underscore the importance of future studies related to better comprehension of the mediatory role of self-esteem through a longitudinal and experimental approach.

## Conclusion

(1) It is important to identify the specific features of the family system as they might constitute significant psychosocial factors associated with EDs.
(2) Apart from FF, dimensions of self-esteem need to be considered in the diagnostic and therapeutic procedures according to their determined mediating role.
(3) EDs among individuals with a negative assessment of FF can be reduced when an individual is satisfied with oneself, has the ability to master new tasks, recognizes the affection received from others, not necessarily the family members, is assertive and persistent, has an established value hierarchy, finds some attractiveness and vitality in themselves, and has their own internal integrity.

## Ethics Statement

In order to protect confidentiality of participants, this research was conducted following the APA Ethics Code Standard 3.08. The study protocol was approved by the ethics committee of Department of Psychology (KB 3/2018), Szczecin University. The respondents were assured of confidentiality of information and gave informed and written consent for their participation in the study.

## Author Contributions

ZK contributed to the ideation of the review, to the search of literature, and wrote the manuscript. MS conducted the statistical analyses and wrote the manuscript. JF contributed to the search of literature, wrote parts of the manuscript and summarized it. AG contributed to the ideation of the review, collected data, and wrote parts of the manuscript.

## Conflict of Interest Statement

The authors declare that the research was conducted in the absence of any commercial or financial relationships that could be construed as a potential conflict of interest.
